# The critical importance of timing of retrieval practice for the fate of nonretrieved memories

**DOI:** 10.1038/s41598-023-32916-7

**Published:** 2023-04-15

**Authors:** Verena M. Kriechbaum, Karl-Heinz T. Bäuml

**Affiliations:** Department of Experimental Psychology, Regensburg University, 93040 Regensburg, Germany

**Keywords:** Psychology, Human behaviour

## Abstract

Retrieval practice performed shortly upon the encoding of information benefits recall of the retrieved information but causes forgetting of nonretrieved information. Here, we show that the forgetting effect on the nonretrieved information can quickly evolve into recall enhancement when retrieval practice is delayed. During a time window of twenty minutes upon the encoding of information, the forgetting effect observed shortly after encoding first disappeared and then turned into recall enhancement when the temporal lag between encoding and retrieval practice was prolonged. Strikingly, recall enhancement continued to emerge when retrieval practice was postponed up to one week. The results illustrate a fast transition from the forgetting of nonretrieved information to recall enhancement. This fast transition is of relevance for daily life, in which retrieval is often selective and delayed.

## Introduction

Retrieval is not a neutral event that just measures the products of a previous learning experience. Rather, retrieval changes memory, as illustrated by the wealth of research establishing that retrieval can improve memory for the retrieved information^1–4^. However, when retrieving encoded information in daily life, retrieval is often selective and only part of the originally encoded information is retrieved—be it in eyewitness testimony situations, educational settings, or many everyday situations, like family conversations over dinner. It is therefore critical to know if retrieval also influences memory for nonretrieved information.

There is evidence that when participants study material and then practice retrieval of a subset of the material, recall of the other, nonretrieved material is often worse than is recall of studied items in the absence of such retrieval practice^5–8^. The finding thus suggests that retrieval can cause forgetting of nonretrieved information, i.e., information that participants during retrieval practice are not asked to retrieve. However, a feature shared by most of the studies demonstrating such retrieval-induced forgetting has been that retrieval practice followed shortly upon study, with a temporal lag of typically one or two minutes between study and retrieval practice. Employing lag intervals of, for instance, one or two days between study and retrieval practice, more recent research reported other results and found retrieval practice to enhance recall of nonretrieved material^9–11^.

To date, the studies suggesting retrieval-induced forgetting and the studies suggesting retrieval-induced recall enhancement represent rather separate research lines that also differ in potentially critical experimental detail^12,13^. It is therefore unclear how exactly the two opposing effects of retrieval practice are related and whether, for instance, the forgetting effect can evolve into recall enhancement when temporal lag between study and retrieval practice is gradually increased from short to longer temporal lag. In such case, the forgetting effect observed shortly after study should first turn into a neutral effect of retrieval practice and then into recall enhancement.

Such transition between the two opposing effects is suggested by a recent view on the effects of retrieval practice^9,14^. This view states that selective retrieval can trigger inhibition and blocking as well as context retrieval processes, each of which can influence recall of nonretrieved information. Inhibition operates to attenuate possible interference from the other, nonretrieved items during retrieval practice, thus reducing recall of these items^5,6,15^. Recall of these items may also be reduced because retrieval practice strengthens the practiced items, which can block recall of the nonretrieved items at test^16–18^. In contrast, context retrieval operates to reactivate study context, which can serve as a retrieval cue and benefit also recall of the nonretrieved items.

Temporal context—the current pattern of activity in an individual`s mind that can be influenced by environmental as well as internal factors—changes gradually over time^19,20^. Because each studied item is associated with the temporal context in which it is shown, context during study and context at test will often differ and context at retrieval thus not be the optimal cue for studied items. However, context during recall changes in response to recall attempts^21,22^: Recall of an item results in partial reactivation of the context that was present when that item was studied, and this retrieved context then serves as a retrieval cue for other items that had a similar context at study, facilitating recall of these items^21–24^.

**Figure 1:**
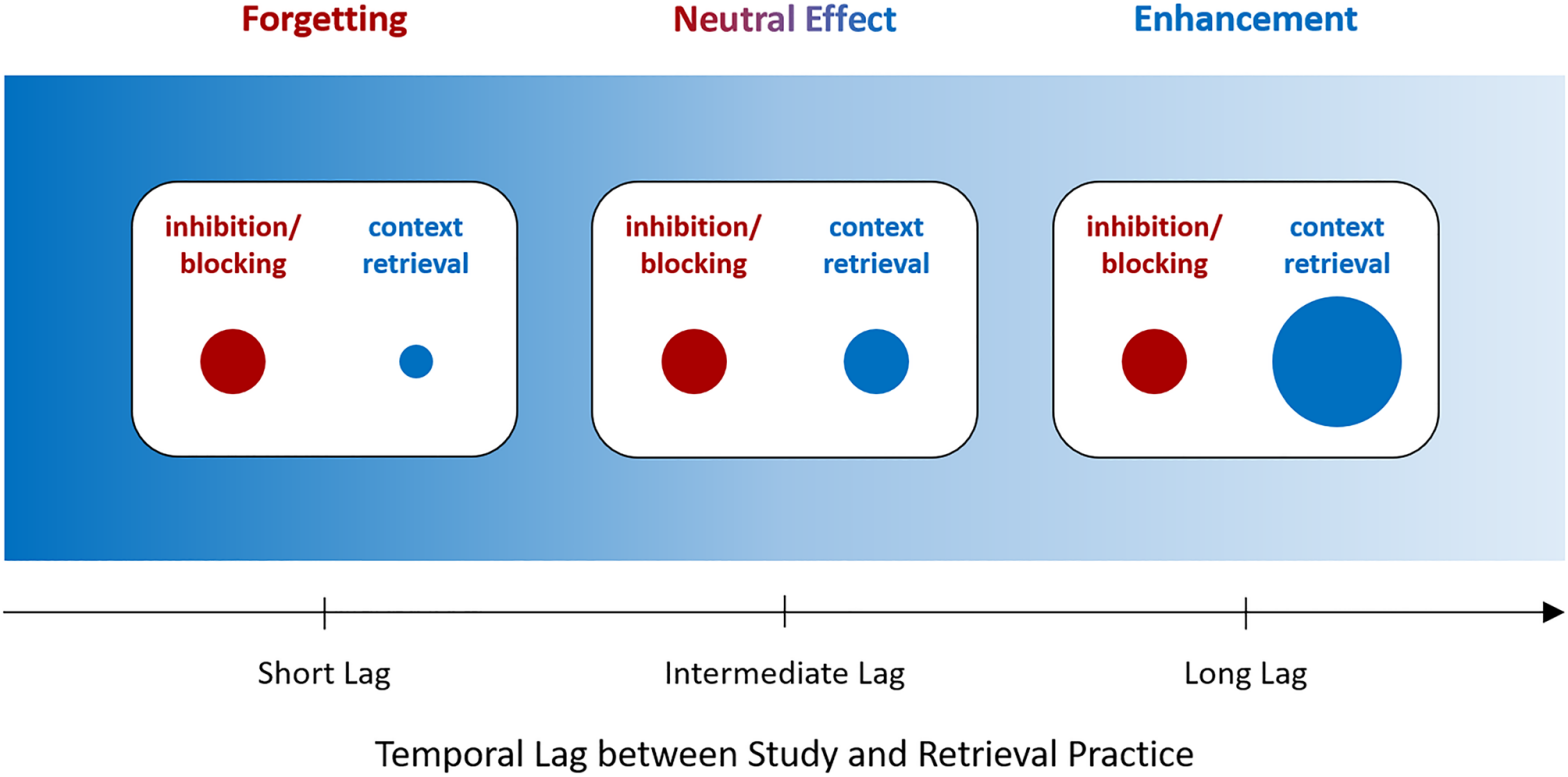
Effects of retrieval practice on the nonretrieved items, i.e., the items that participants during retrieval practice were not asked to retrieve. Hypothetical relative contributions of inhibition, blocking, and context retrieval to recall of the nonretrieved items are shown as a function of temporal lag between study and retrieval practice. After short lag—when temporal context is still similar to study context—the relative contributions of inhibition and blocking are high and that of context retrieval is low, inducing forgetting of the nonretrieved items. When temporal lag increases—and temporal context gets dissimilar to study context—the contribution of context retrieval also increases, which turns the forgetting effect into a neutral and then an enhancement effect on recall of the nonretrieved items.

Critically, the relative contribution of context retrieval to recall should be small shortly after study when temporal context is still similar to study context, but it should increase as temporal lag between study and retrieval practice increases and temporal context becomes dissimilar to study context (Fig. 1). A gradually increasing lag between study and retrieval practice may thus induce a transition from the forgetting effect caused by inhibition and blocking shortly after study into a neutral effect and then the enhancement effect of retrieval practice. It is the primary goal of this study to demonstrate such transition, which will also provide critical information on how narrow the time window after study is during which retrieval produces forgetting and what the time frame is during which retrieval produces recall enhancement. Such information will impose important restrictions on theories of memory retrieval and create suggestions on how selective retrieval influences memory in daily life, be it in eyewitness testimony or educational situations.

**Figure 2:**
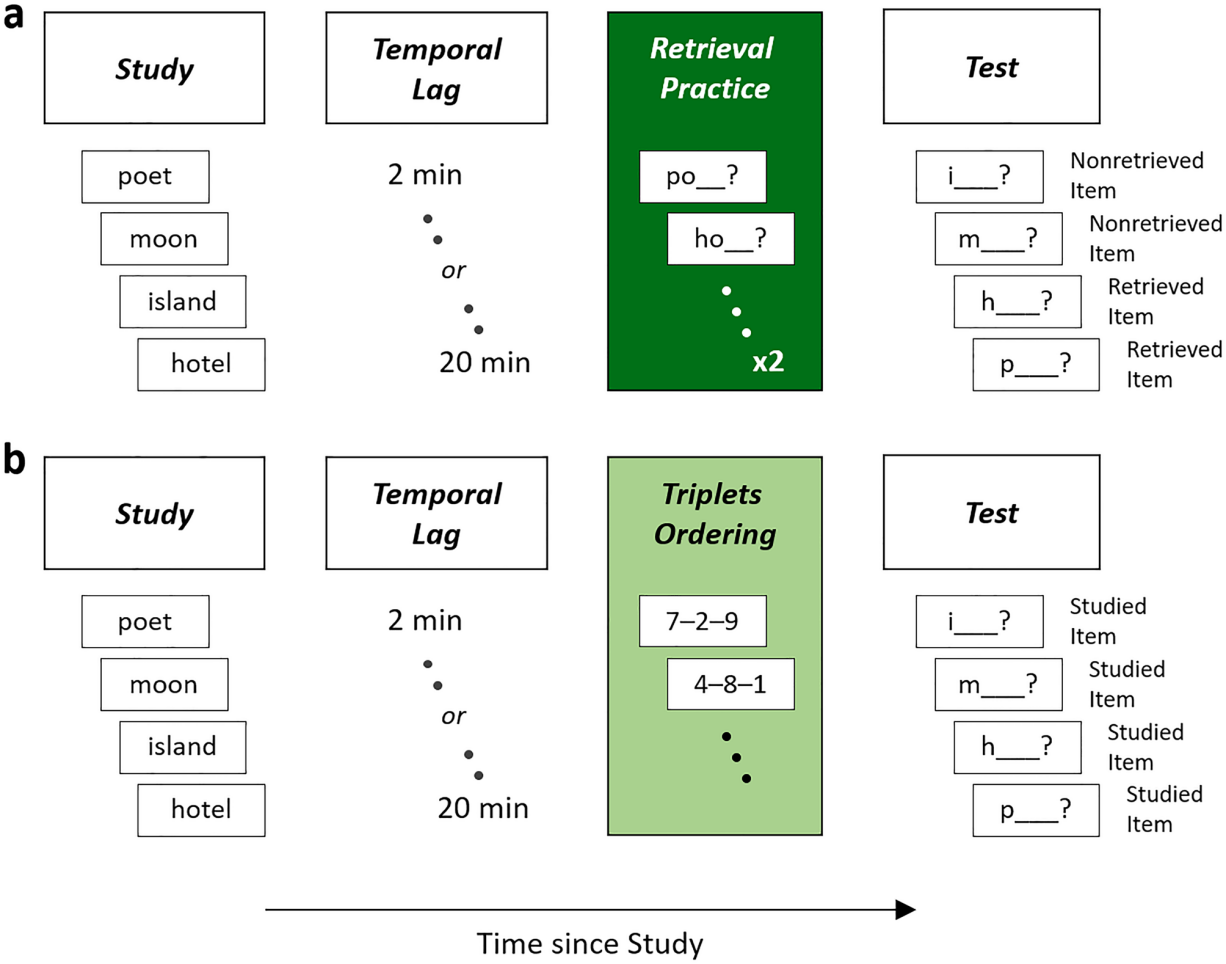
Experimental design for experiments 1 and 2. Two groups of participants studied a list of words. (**a**) Recall of one group was tested after retrieval practice of some of the items, which took place after temporal lags of 2, 8, 14, or 20 min after study in experiment 1, and after temporal lags of 2, 11, or 20 min after study in experiment 2, and created retrieved and nonretrieved items. Different subgroups of the group were tested in the different lag conditions. (**b**) Recall of the other group was tested in the absence of retrieval practice after a triplets ordering task serving as a control. Different subgroups of the group engaged in the task 2, 8, 14, or 20 min after study in experiment 1, and 2, 11, or 20 min after study in experiment 2.

Here, results from two experiments are reported aimed at shedding light onto whether the forgetting effect of retrieval practice transforms into recall enhancement when temporal lag between study and retrieval practice is gradually increased from short to longer lag interval. In both experiment 1 and experiment 2, recall of nonretrieved items after retrieval practice was compared with recall of studied items when a triplets ordering task serving as a control rather than retrieval practice preceded the recall test (Fig. 2). During retrieval practice, some studied items were retrieved, creating retrieved and nonretrieved items, i.e., items that participants during retrieval practice were not asked to retrieve. During the triplets ordering task, participants were presented number triplets and were asked to order each triplet from highest to lowest number. Recall of studied and nonretrieved items was compared for a short 2-min and a longer 20-min lag between study and retrieval practice as well as two intermediate (experiment 1) or one intermediate (experiment 2) lag interval(s). Results from a third experiment are also reported investigating whether the enhancement effect of retrieval practice still emerges when lag interval is prolonged up to one whole week.

## Results

### Experiments 1 and 2

In each experiment, participants studied a list of items and were later tested on the list. Participants were divided into two groups to understand how retrieval practice influences recall of the nonretrieved items. Recall of the one group was tested after preceding retrieval practice, which took place 2, 8, 14, or 20 min after study in experiment 1, and 2, 11, or 20 min after study in experiment 2. Different subgroups were tested in the different lag conditions. Recall of the other group was tested in the absence of retrieval practice. The group was also divided into different subgroups and each subgroup engaged into the triplets ordering (distractor) task 2, 8, 14, or 20 min after study in experiment 1, and 2, 11, or 20 min after study in experiment 2. After retrieval practice, and after the triplets ordering task, participants were tested on the initially encoded items. During the lag intervals, participants engaged in cognitive (distractor) tasks that were unrelated to the memory task. Different sets of tasks were used in the two experiments, each task being similar to tasks used in prior work on retrieval practice effects (Supplementary Information).

**Figure 3:**
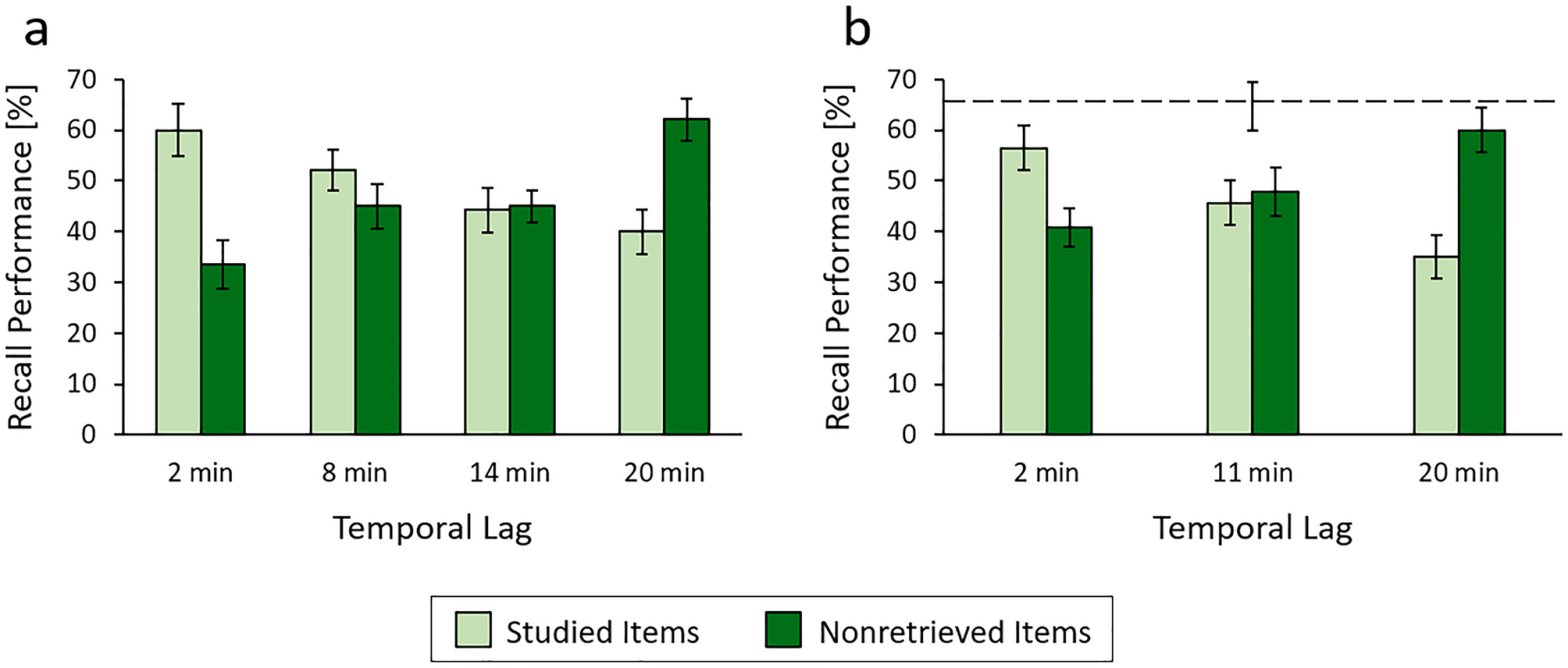
Results of experiment 1 (**a**) and experiment 2 (**b**). Recall of the studied items decreased but recall of the nonretrieved items increased from the shorter to the longer temporal lag conditions. After the short 2-min lag, recall of the studied items was superior to recall of the nonretrieved items; after the longer 20-min lag, the pattern reversed and recall of the nonretrieved items was superior to recall of the studied items; recall of the two item types was similar in the intermediate lag conditions. In experiment 2, recall of the nonretrieved items after the 20-min lag resembled recall of studied items when the studied items were tested immediately after study (indicated by the dashed line in (**b**). Error bars represent ± 1 SE.

In all three experiments, variance of recall rates did not differ across conditions, as indicated by the results of Levene`s tests. This held when analyzing the effects of temporal lag and item type for the nonretrieved and the studied items (experiment 1: *P* = 0.190, experiment 2: *P* = 0.890, experiment 3: *P* = 0.190) and when analyzing the effects of temporal lag for the retrieved items (experiment 1: *P* = 0.723, experiment 2: *P* = 0.367, experiment 3: *P* = 0.452). We therefore employed analysis of variance and post-hoc t-tests to analyze how recall rates varied across conditions. In experiment 1, typical time-dependent forgetting emerged for the studied items in the absence of retrieval practice, with recall of the items declining from the short to the longer lag conditions, whereas the opposite pattern was present after retrieval practice, with recall of the nonretrieved items increasing as the lag interval increased (Fig. 3a). Consistently, a two-way analysis of variance with the between-participants factors of lag condition and item type revealed no main effect of lag condition (*F*(3, 216) = 0.79, *P* = 0.501, *η*^2^ = 0.01) and no main effect of item type (*F*(1, 216) = 0.76, *P* = 0.384, *η*^2^ < 0.01), but a significant interaction between the two factors (*F*(3, 216) = 10.73, *P* < 0.001, *η*^2^ = 0.13). Critically, retrieval practice impaired recall of the nonretrieved items relative to recall of the studied items after the short 2-min lag (two-tailed t-test: *t*(54) = 3.75, *P*_adj_ = 0.004, *d* = 1.00, 95% CI of the difference = [− 40.58, − 12.28]), but it improved recall of the nonretrieved items after the longer 20-min lag (two-tailed t-test: *t*(54) = 3.67, *P*_adj_ = 0.003, *d* = 0.98, 95% CI of the difference = [10.06, 34.23]). In the intermediate lag conditions, no effects of retrieval practice arose (8-min condition, two-tailed t-test: *t*(54) = 1.21, *P*_adj_ = 0.464, *d* = 0.32, 95% CI of the difference = [− 4.70, 18.98], B_01_ = 3.56; 14-min condition, two-tailed t-test: *t*(54) = 0.13, *P*_adj_ = 0.896, *d* = 0.04, 95% CI of the difference = [− 11.62, 10.20], B_01_ = 7.39). To control the familywise error rate across the four comparisons, *P*-values were adjusted by employing the sequential Bonferroni procedure.

In experiment 2, recall of the studied items again decreased and recall of the nonretrieved items again increased from the short to the longer lag conditions (Fig. 3b), thus mimicking recall of the two item types in experiment 1. Again, a two-way analysis of variance with the between-participants factors of lag condition and item type revealed no main effect of lag condition (*F*(2, 162) = 0.09, *P* = 0.919, *η*^2^ < 0.01) and no main effect of item type (*F*(1, 162) = 1.14, *P* = 0.287, *η*^2^ < 0.01), but a significant interaction between the two factors (*F*(2, 162) = 10.90, *P* < 0.001, *η*^2^ = 0.12). Critically, retrieval practice impaired recall of the nonretrieved items relative to recall of the studied items after the short 2-min lag (two-tailed t-test: *t*(54) = 2.72, *P*_adj_ = 0.018, *d* = 0.73, 95% CI of the difference = [− 27.30, − 4.13]), but it improved recall of the nonretrieved items after the longer 20-min lag (two-tailed t-test: *t*(54) = 4.02, *P*_adj_ = 0.003, *d* = 1.07, 95% CI of the difference = [12.52, 37.48]). In the intermediate lag condition, no effect of retrieval practice arose (11-min condition, two-tailed t-test: *t*(54) = 0.33, *P*_adj_ = 0.744, *d* = 0.09, 95% CI of the difference = [− 15.22, 10.93], B_01_ = 7.10). Like in experiment 1, the *P*-values for all three comparisons were adjusted by using the sequential Bonferroni procedure. In both experiment 1 and experiment 2, the forgetting induced by retrieval practice after short lag thus first turned into a neutral effect of retrieval practice, and then into recall enhancement as temporal lag was increased from 2 to 20 min. The transition was largely unaffected by the different cognitive tasks participants engaged in during the lag intervals in experiments 1 and 2.

The fact that retrieval practice enhanced recall of the nonretrieved items relative to the studied items after the 20-min lag implies that retrieval practice attenuated the items' time-dependent forgetting. To provide insight into whether retrieval practice even eliminated the items' forgetting over time, in experiment 2, recall of the nonretrieved items after the 20-min lag was compared to recall of studied items when these items were tested immediately after study in the absence of retrieval practice and in the absence of the triplets ordering task. Recall of the nonretrieved items was similar to recall in this immediate recall condition (two-tailed t-test: *t*(54) = 0.88, *P* = 0.383, *d* = 0.24, 95% CI of the difference = [− 7.31, 18.74], B_01_ = 5.01), suggesting that retrieval practice largely eliminated the items' forgetting over time.

In contrast to the studied and the nonretrieved items, recall of the retrieved items did not vary with temporal lag (experiment 1, one-way ANOVA: *F*(3, 108) = 0.10, *P* = 0.961, *η*^2^ < 0.01; experiment 2, one-way ANOVA: *F*(2, 81) = 0.31, *P* = 0.733, *η*^2^ < 0.01) and thus followed the items' recall during retrieval practice (Supplementary Information, Supplementary Tables 1–2).

### Experiment 3

The fact that retrieval practice largely eliminated nonretrieved items' forgetting over time when retrieval practice occurred 20 min after study suggests that retrieval practice reinstated study context more or less completely, thus making recall after retrieval practice comparable to recall directly after study. However, reinstating study context may get harder if the lag interval between study and retrieval practice is increased up to hours or even days. For such prolonged lag intervals, only part of the accumulated time-dependent forgetting may therefore be eliminated. Using similar experimental setup as was employed in experiments 1 and 2, experiment 3 was aimed at examining the effects of retrieval practice for lag intervals of 2 h, 2 days, and 7 days. In all three lag conditions, recall of the nonretrieved items after retrieval practice was compared to recall of the studied items when participants were engaged in the triplets ordering task prior to the recall test. Following experiment 2, recall of the nonretrieved items was also compared to recall of studied items when these items were tested immediately after study in the absence of retrieval practice and in the absence of the triplets ordering task.

**Figure 4:**
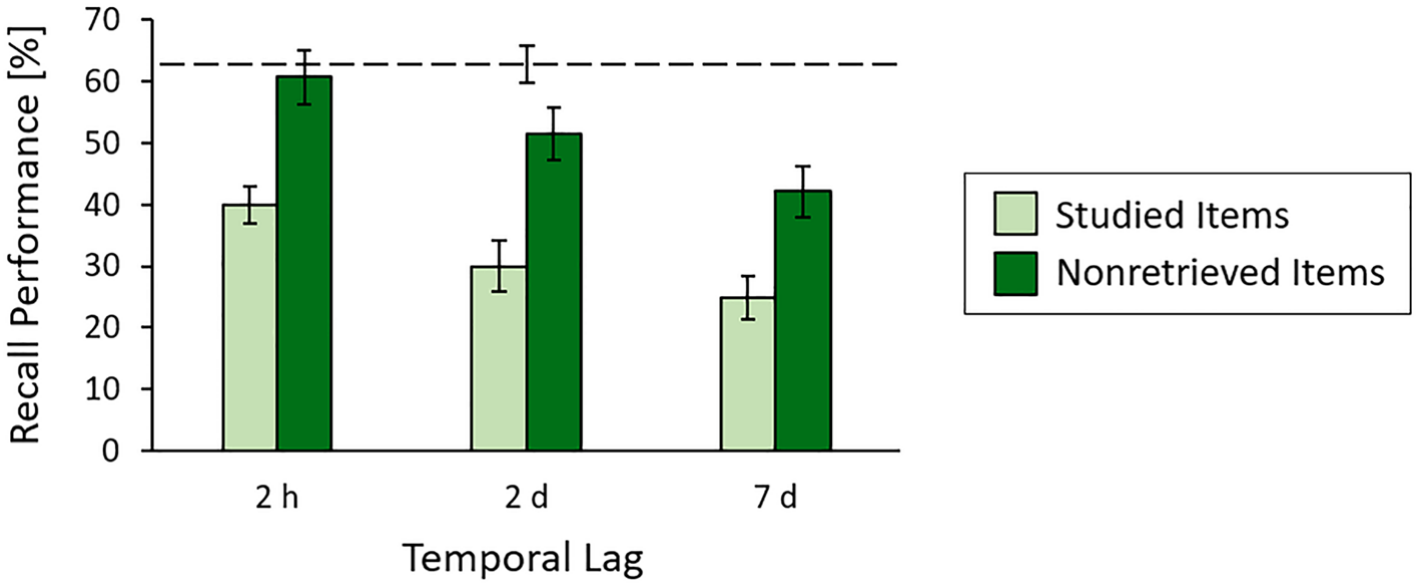
Results of experiment 3. Both recall of the studied items and recall of the nonretrieved items decreased with increasing temporal lag. In all three lag conditions, recall of the nonretrieved items was superior to recall of the studied items. Recall of the nonretrieved items after the 2-h lag resembled recall of studied items when the studied items were tested immediately after study (indicated by the dashed line), whereas, after the 2-d and 7-d lags, recall of the nonretrieved items was inferior to recall in this immediate recall condition. Error bars represent ± 1 SE.

For both the studied items and the nonretrieved items, typical time-dependent forgetting emerged, with recall of the nonretrieved items after retrieval practice being superior to recall of the corresponding studied items (Fig. 4). A two-way analysis of variance with the between-participants factors of lag condition and item type found main effects of lag condition (*F*(2, 162) = 9.00, *P* < 0.001, *η*^2^ = 0.10) and item type (*F*(1, 162) = 37.16, *P* < 0.001, *η*^2^ = 0.19), but no significant interaction between the two factors (*F*(2, 162) = 0.17, *P* = 0.846, *η*^2^ < 0.01), suggesting that retrieval practice can enhance recall of the nonretrieved items also after temporal lags of hours and even days and does so to a similar degree across conditions. In the 2-h and 2-d lag conditions, recall of the nonretrieved items was even similar to recall in the immediate recall condition (2-h condition, two-tailed t-test: *t*(54) = 0.40, *P*_adj_ = 0.691, *d* = 0.11, 95% CI of the difference = [− 8.61, 12.90], *B*_01_ = 6.93; 2-d condition, two-tailed t-test:* t*(54) = 2.17, *P*_adj_ = 0.068, *d* = 0.58, 95% CI of the difference = [− 21.97, − 0.88], B_01_ = 0.71), thus mimicking results in the 20-min lag condition of experiment 2 and indicating that, also after hours, retrieval practice can largely eliminate nonretrieved items' forgetting over time. In contrast, in the 7-d lag condition, recall of the nonretrieved items was inferior to recall in the immediate recall condition (two-tailed t-test: *t*(54) = 4.02, *P*_adj_ = 0.003, *d* = 1.08, 95% CI of the difference = [− 31.04, − 10.39]), suggesting that retrieval practice eliminated only part of the accumulated time-dependent forgetting. For all three comparisons of the nonretrieved items' recall rates to the immediate recall condition, *P*-values were again adjusted following the sequential Bonferroni procedure. With the larger range of lag intervals employed in this experiment relative to experiments 1 and 2, recall of the retrieved items also decreased with temporal lag (one-way ANOVA: *F*(2, 81) = 9.75, *P* < 0.001, *η*^2^ = 0.19). Like in experiments 1 and 2, recall of the retrieved items at test resembled the items’ recall during retrieval practice (Supplementary Information, Supplementary Table 3).

## Discussion

This study demonstrates that the forgetting that retrieval practice produces for the nonretrieved material when it occurs shortly upon study can evolve into recall enhancement. The observed forgetting first turns into a neutral effect of retrieval practice and then into recall enhancement if the temporal lag between study and retrieval practice gradually increases from short to longer lag interval. Prior work had already demonstrated enhancement effects on the nonretrieved items after longer lag^9–11,23^, but retrieval practice was mostly part of the test phase in these studies and recall of studied items was measured in the presence versus absence of the preceding recall ("retrieval practice") of other studied items. This study shows that the enhancement effect also arises when retrieval practice and test are separated into distinct experimental phases and, while participants in the retrieval-practice condition engage in retrieval practice, participants in the no-retrieval-practice condition engage in an unrelated cognitive task of equal duration as a control, which has become the standard paradigm to study the forgetting effect of retrieval practice^8,12,25^.

The results illustrate that the transition from retrieval-produced forgetting into recall enhancement can be fast. Typical forgetting of the nonretrieved items emerged when retrieval practice occurred 2 min after study, but the forgetting quickly disappeared when temporal lag between study and retrieval practice was increased. Recall of the nonretrieved items was more or less unaffected by retrieval practice when practice took place about 10 min after study, and another 10 min later, retrieval practice already led to recall enhancement. Strikingly, the recall enhancement observed 20 min after study was sufficiently strong to eliminate the time-dependent forgetting that had accumulated since study. Retrieval practice thus effectively protected the nonretrieved items from showing forgetting over time.

These findings are consistent with the idea that context retrieval critically contributes to recall when retrieval practice is delayed^9,14,24^. Shortly upon study, when temporal context is still similar to study context, recall can not benefit much from context retrieval but inhibition and blocking operate in response to retrieval practice, which causes forgetting of the nonretrieved items. However, as time after study passes and context gets more and more dissimilar to study context, recall benefits from context reactivation and context retrieval enhances recall of the nonretrieved items. Critically, the finding that 20 min after study retrieval practice eliminated the time-dependent forgetting that had accumulated since study does not only suggest that study context reactivation was more or less complete, it also indicates that inhibition and blocking barely contributed to recall at this point in time. Retrieval practice therefore caused mainly inhibition and blocking shortly after study, and mainly context retrieval about 20 min later.

The finding that, in both experiment 1 and experiment 2, the beneficial effect of retrieval practice arose when retrieval practice was delayed by 20 min shows that the difference in cognitive (distractor) tasks between the two experiments did not much influence the results. Still, type of distractor task may affect results. For instance, if participants were engaged in daydreaming distractor tasks, which have been shown to enhance internal context change^26,27^, context retrieval may play a stronger role for recall than it did here and the beneficial effect of retrieval practice thus arise even earlier^28^. Item lists may also influence results. For instance, if item lists were presented to participants that induced a higher level of interitem interference than the lists employed here, the amount of blocking and inhibition on the nonretrieved items may be enhanced and the beneficial effect arise somewhat later. Future work is required to pin down the range of possible cross-over points between the detrimental and beneficial effects as well as the range of possible lag intervals after which the beneficial effect arises. Likely, such results will show that, in general, the beneficial effect emerges after rather short lag between study and retrieval practice, a lag interval in the order of minutes, not of hours or even days.

The results also reveal that context retrieval contributed to recall when retrieval practice took place 2 h, 2 days, and even 7 days after study, again enhancing recall of the nonretrieved items. However, whereas retrieval practice again protected the nonretrieved items from showing forgetting over time when it occurred 2 h after study, only part of the time-dependent forgetting was eliminated when lag interval increased to seven days, indicating that study context reactivation can become incomplete after very long lag^23,29^. The change from complete to incomplete elimination of time-dependent forgetting was accompanied by a reduction in recall success during retrieval practice (Supplementary Information), which fits with the view that recall success during retrieval practice is a critical component for successful study context reactivation^23^.

Study context can not only be reactivated through retrieval practice. Context reactivation can also arise if participants, some time after study, are asked to mentally reinstate study context^23^. Such deliberate active reinstatement attempts can make recall superior relative to a no-reinstatement condition, although, often, they do not lead to perfect study context reactivation^26,30,31^. Individuals also can maintain and use an older context when they know the task requires so. If individuals learn a series of lists of items and, from second list on, are asked after each list to recall the list prior to the last presented list, they are able to use the context that is appropriate for the prior list rather than the current one, though experience with the task may be required to show the effect^32,33^.

The present experiments varied the lag between study and retrieval practice while holding the delay between retrieval practice and test constant and short, which allows to measure possible effects of blocking, inhibition, and context retrieval on the nonretrieved items more or less directly after practice. The present research thus differs from research on the so-called spacing effect, in which the beneficial mnemonic effect of spaced over massed practice on studied material is examined while holding the retention interval between study and test constant^34–36^. Future work may thus bridge the gap between the present research and research on the spacing effect by examining the effects of time-lagged selective retrieval also for constant retention interval.

A number of studies have identified neural correlates of inhibition as induced by retrieval practice. The studies provided evidence for critical roles of both the anterior cingulate cortex and the lateral prefrontal cortex and indicated that retrieval practice indeed suppresses the nonretrieved items' memory representations^37–39^. Lateral prefrontal cortex has also been identified as a possible correlate of blocking processes^40^. Studies investigating neural correlates of context retrieval are relatively scarce to date but suggested roles of lateral prefrontal cortex as well as medial and lateral parietal lobe regions^21,41,42^. This study offers an experimental setup that may be applied to investigate neural correlates of inhibition and blocking as well as context retrieval when the two types of processes operate mainly in isolation and when they operate in concert. With temporal lag between study and retrieval practice as the critical factor, lags of about 2 min may be used to study inhibition and blocking when context retrieval is more or less absent and lags of at least 20 min may be used to study context retrieval when inhibition and blocking are largely absent. Intermediate temporal lags may reveal possible interactions between the two types of processes.

Retrieval practice after short lag does not always produce forgetting. Coherent study material, for instance, can reduce interference between the single memory entries and make inhibition and blocking obsolete^43,44^. In such cases, retrieval practice triggers mainly context retrieval and may improve recall of the nonretrieved material if retrieval practice is performed only few minutes after encoding of the memory entries. Similarly, retrieval practice after longer lag will not always produce recall enhancement. If particularly salient features surrounded an encoded episode, reactivation of such features—be it through reexposure of the features or deliberate active reinstatement attempts—immediately before retrieval practice starts may revive the encoding context and thus reduce the likelihood of further retrieval-induced context retrieval^23,45^. Thus, not only lag between study and retrieval practice but also a few other factors can influence whether retrieval practice produces forgetting or recall enhancement for other memory contents.

The experimental task employed here as well as the experimental tasks used in prior work^23,24^ show some specific features. For instance, during retrieval practice, two-thirds of the studied material are practiced and practice is conducted in two successive practice cycles, features that may enhance the effects of retrieval practice. Or, at test, item-specific retrieval cues are presented and the nonretrieved items are tested before the retrieved items, features that permit rather direct measurement of nonretrieved items’ blocking, inhibition, or context reactivation. Likely, the size of the effects of retrieval practice would decrease only slightly if a smaller proportion of the studied material was practiced or a single practice cycle was conducted only^8–10^. Whether testing the retrieved items first and the nonretrieved items last or an alternative free recall format—in which the (stronger) retrieved items would also tend to be recalled first—would influence results is less clear, but prior recall of the retrieved items could serve as an additional opportunity for blocking, inhibition, and context reactivation and thus potentially increase the effects on the nonretrieved items.

Retrieval practice can trigger inhibition and blocking and cause forgetting of nonretrieved information. This study shows that, when retrieval practice is delayed, it can also trigger context retrieval that reactivates the encoding context and enhances recall of the nonretrieved information. Critically, the transition between forgetting and recall enhancement can be fast. During a time window of twenty minutes upon encoding, the forgetting observed shortly after study first disappeared and then turned into recall enhancement as temporal lag between study and retrieval practice was increased. Strikingly, recall enhancement continued to emerge when retrieval practice was postponed by several days or even one whole week. The findings are of high relevance for daily life, because in the real world retrieval is often selective and it is often delayed. In such situations, retrieval practice may be an effective tool to improve also recall of other, nonretrieved memories.

## Methods

### Experiment 1

*Participants.* The participants (224 students of different German universities, mean age 23.61 y, 75.9% females) were divided into two groups, each consisting of four subgroups of *n* = 28 participants. Sample size was determined on the basis of a power analysis^46^ using alpha = 0.05 and beta = 0.20 and effect sizes of *d* = 0.80 for expected time-dependent forgetting and expected detrimental and beneficial effects of retrieval practice^9,10,23,24,47^. The participants were tested individually in an online video conference hosted by the software Zoom (Zoom Video Communications, 2016). Instructions were given by the experimenter, who was present for the entire period of the experiment.

*Materials.* A list of 15 unrelated concrete German nouns was employed as study material^23,24^. Each item had a unique initial letter. The items served as studied items when retrieval practice was absent and as retrieved and nonretrieved items when retrieval practice was present. Ten items of the list served as the retrieved items and the other five items served as the nonretrieved items. Within each lag condition, each item was a retrieved item for *n* = 18 or *n* = 19 participants and a nonretrieved item for *n* = 9 or *n* = 10 participants.

*Procedure.* Each participant in this experiment—as well as in experiments 2 and 3—provided informed consent prior to participation. The protocol employed in this study was reviewed and deemed exempt by the ethical review board of Regensburg University. The experiments were carried out in accordance with the provisions of the World Medical Association Declaration of Helsinki. During study, the items of the list were presented individually and in a random order for 6 s each on the computer screen. Four different lag intervals (2, 8, 14, and 20 min) followed, filled with cognitive tasks that were unrelated to the memory task, including mental rotation of dices, applied arithmetics, and detecting repetitions of stimulus features in a sequence of visually presented objects (Supplementary Information). In each single lag condition, half of the participants then engaged in retrieval practice, whereas the other half engaged in a triplets ordering task. During retrieval practice, participants were asked to recall 10 of the 15 items (the retrieved items). The items' first two letters served as retrieval cues and were presented in a random order for 6 s each. There were two rounds of practice. During the triplets ordering task, participants were presented number triplets for the equivalent amount of time. They were asked to order each triplet from highest to lowest number. After a subsequent 2-min counting task, all participants were finally asked to recall all 15 items. The items' initial letters served as retrieval cues and were presented in a random order for 6 s each. Order of tested items was random but, in the retrieval practice group, the nonretrieved items were always tested first and the retrieved items last^5,12,24,25^. All responses given by the participants in this experiment were given orally. Because recall performance can vary with items’ output position at test^48–50^, we followed prior work on the effects of selective retrieval practice and compared recall rates of the nonretrieved items with recall rates of studied items tested in the same—i.e., first five—output positions^5,24^.

### Experiment 2

*Participants.* Another sample of participants (196 students of different German universities, mean age 24.1 y, 79.1% females) was divided into three groups. Two of the groups engaged in retrieval practice or the triplets ordering task and consisted of three subgroups of *n* = 28 participants each. The third group (*n* = 28) did not engage in retrieval practice or triplets ordering.

*Materials.* Another list of 15 unrelated concrete German nouns was employed as study material ^23,24^. Again, the items had unique initial letters. The division of the items into studied, retrieved, and nonretrieved items followed experiment 1.

*Procedure.* The procedure differed in three aspects from experiment 1: (1) The lag intervals after study were changed to 2, 11, and 20 min; (2) a different set of cognitive tasks was employed to fill the single lag intervals, including mental overlaying of visual objects, the operation span task, a progressive matrices test, and a fill-in-the-arithmetic-operators task (Supplementary Information); (3) there was an immediate recall condition, which was identical to the other six conditions with regard to study and test but differed from these conditions in that no retrieval practice and no triplets ordering was conducted; rather, recall was tested directly after study and a 2-min distractor task (and thus 4 min earlier than in the 2-min lag conditions, in which the 2-min lag was followed by 2 min of retrieval practice or triplets ordering and another 2-min distractor task).

### Experiment 3

*Participants.* Like in experiment 2, a distinct sample of participants (196 students of different German universities, mean age 24.0 y, 69.4% females) was divided into three groups. Two groups engaged in retrieval practice or the triplets ordering task and consisted of three subgroups of *n* = 28 participants each. The third group (*n* = 28) did not engage in these tasks.

*Materials.* The same material was used as in experiment 2. The division of the items into studied, retrieved, and nonretrieved items followed experiment 1.

*Procedure.* The procedure differed in one aspect from experiment 2: the lag intervals after study were changed to 2 h, 2 d, and 7 d. In all three lag conditions, a 2-min distractor task followed study and the participants were then dismissed for this lag interval, rejoining the experiment later. The same immediate recall condition was included as in experiment 2.

## Supplementary Information

Supplementary Information.

## Data Availability

The data from the single experiments as well as the materials employed in the experiments are available on the Open Science Framework (https://osf.io/x5e3r/?view_only=67ff5f35e12b4b7e80a14b1b71694dba).

## Code availability

All experiments reported in this manuscript were implemented using the software PowerPoint 2019 (Microsoft Corporation) and the software Zoom (Zoom Video Communications Inc., 2016). The software was run on standard desktop computers with the operating system Windows 10 (Microsoft, Redmond, WA). Data were analyzed using IBM SPSS Statistics for Windows, Version 27.0 (IBM Corp., Armonk, NY) and G*Power 3.1^46^.
