# Supplementary material for: The critical importance of timing of retrieval practice for the fate of nonretrieved memories
Source: Sci Rep. 2023 Apr 15;13:6128. doi: 10.1038/s41598-023-32916-7 (PMC10105692; doi:10.1038/s41598-023-32916-7)
Supplement: Supplementary file 1 — Supplementary Information. [file 41598_2023_32916_MOESM1_ESM.pdf]

Supplementary Information for

The Critical Importance of Timing of Retrieval Practice for the Fate of Nonretrieved Memories

Verena M. Kriechbaum, Karl-Heinz T. Bäuml

Department of Experimental Psychology, Regensburg University, Germany

Correspondence Address:

Karl-Heinz T. Bäuml,

Department of Experimental Psychology, Regensburg University, 93040 Regensburg, Germany.

Phone: +49 941 943-3818

Email: karl-heinz.baeuml@ur.de

This PDF file includes:

Supplementary Information
- Recall of Retrieved Items during Retrieval Practice and at Test
- Cognitive (Distractor) Tasks

Supplementary Tables 1 to 3
Supplementary References

Supplementary Information

Recall of Retrieved Items during Retrieval Practice and at Test. Supplementary Tables 1–3 show recall of retrieved items during retrieval practice and at test. As described in the Main Manuscript, recall of retrieved items at test was unaffected by lag in experiments 1 and 2 but decreased with increasing lag in experiment 3. The difference in findings reflects the fact that experiment 3 employed a much wider range of lag intervals (nearly seven days) than did experiments 1 and 2 (less than 20 minutes).

Recall of retrieved items during retrieval practice showed the same pattern of results as recall of retrieved items at test. Because, in all three experiments, variances of recall rates did not differ across conditions (Levene`s tests: all *P*s > .127), we conducted an analysis of variance to compare recall rates across conditions. In experiments 1 and 2, recall during retrieval practice was unaffected by lag condition (experiment 1, mixed-factorial ANOVA: $F(3, 108) = 1.31$, $P = .275$, $\eta^2 = .04$; experiment 2, mixed-factorial ANOVA: $F(2, 81) = 0.29$, $P = .748$, $\eta^2 < .01$), whereas in experiment 3, recall during retrieval practice decreased with increasing lag (mixed-factorial ANOVA: $F(2, 81) = 7.91$, $P < .001$, $\eta^2 = .16$). In all three experiments, there was a main effect of retrieval practice cycle, with recall increasing from the first to the second retrieval practice cycle (all *P*s < .001), but there was no interaction between lag condition and retrieval practice cycle (all *P*s > .132).

In all three experiments, mean recall of the retrieved items was similar between second retrieval practice cycle and test (experiment 1: 64.9% vs. 61.9%; experiment 2: 74.5% vs. 71.0%; experiment 3: 66.3% vs. 67.0%). This holds although, during retrieval practice, the first two letters of each item served as a retrieval cue, and, at test, only the initial letter of each single item was provided as cue. The similarity in recall levels between retrieval practice and test reflects the fact that there was only a short 2-min delay between retrieval practice and test, and recall of retrieved items at test thus could benefit from preceding recall of the very same items during retrieval practice.

A comparison of recall levels between retrieved items (Supplementary Tables 1–3) and nonretrieved items (Figures 3–4) shows that, in nine of the ten experimental conditions, recall of the retrieved items exceeded recall of the nonretrieved items, with differences between 10% and 30% between the two item types. Only in the 20-min condition of experiment 1, recall levels of retrieved and nonretrieved items were roughly equal (61.07% vs. 62.14%).

Cognitive (Distractor) Tasks. In experiment 1, besides simple counting backwards, three neutral distractor tasks were employed to fill the single lag intervals. Immediately after study, all participants counted backwards for 2 min, which filled the lag interval in the 2-min lag condition. In the 8, 14, and 20-min lag conditions, participants engaged in one, two, or three further distractor tasks. Each distractor task lasted for 6 min and was sampled randomly (without replacement) from i) mental rotation of dices [1], ii) applied arithmetics [1], and iii) detecting repetitions of stimulus features in a sequence of visually presented objects (http://brainworkshop.sourceforge.net).

In experiment 2, besides simple counting backwards, another four neutral distractor tasks were employed to fill the single lag intervals. Immediately after study, all participants counted backwards for 2 min, which filled the lag interval in the 2-min lag condition. In the 11-min lag condition, one block of two further distractor tasks followed, and, in the 20-min lag

condition, two blocks, each with two further distractor tasks, followed. Each block lasted for 9 min and consisted either of i) mental overlaying of visual objects [2] followed by the operation span task [3], or ii) a progressive matrices test [4] followed by a fill-in-the-arithmetic-operators task [4]. The pair of tasks that was employed for a block was sampled randomly (without replacement) from the two possible pairs of tasks.

Both the distractor tasks employed in experiment 1 and the distractor tasks employed in experiment 2 are similar to distractor tasks used in prior research on retrieval practice effects, both testing effect studies [5, 6] and studies on retrieval-induced forgetting [7, 8] and retrieval-induced enhancement [9, 10]. There are no results in the literature on whether the single distractor tasks differ much in the degree to which they induce internal context change. Accordingly, there was no way to create any reasonable expectations on whether results would vary with employed distractor tasks.

Supplementary Table 1.
Recall performance [%] of retrieved items in experiment 1 as a function of temporal lag between study and retrieval practice (2 min, 8 min, 14 min, 20 min) during first retrieval practice cycle, during second retrieval practice cycle, and at test.

	2 min	8 min	14 min	20 min
1st RPC	66.07	56.43	57.86	62.50
2nd RPC	70.00	62.14	61.43	66.07
Test	63.57	61.79	61.07	61.07

Note. RPC = retrieval practice cycle.

Supplementary Table 2.
Recall performance [%] of retrieved items in experiment 2 as a function of temporal lag between study and retrieval practice (2 min, 11 min, 20 min) during first retrieval practice cycle, during second retrieval practice cycle, and at test.

	2 min	11 min	20 min
1st RPC	71.79	67.50	71.79
2nd RPC	76.07	73.57	73.93
Test	69.64	70.00	73.21

Note. RPC = retrieval practice cycle.

Supplementary Table 3.
Recall performance [%] of retrieved items in experiment 3 as a function of temporal lag between study and retrieval practice (2 h, 2 d, 7 d) during first retrieval practice cycle, during second retrieval practice cycle, and at test.

	2 h	2 d	7 d
1st RPC	73.93	58.93	53.57
2nd RPC	77.14	62.86	58.93
Test	78.21	65.36	57.50

Note. RPC = retrieval practice cycle.

Supplementary References

1. Ibrahimović, N., Bulheller, S., Horn, R., Gittler, G. & Institut für Test- und Begabungsforschung GmbH. *IBF: Intelligenz-Basis-Faktoren* (Harcourt, Frankfurt am Main, 2006).
2. Dantlgraber, M., Hell, B., Bossinger-Fischer, F. & Schult, J. *M-KIT: Modularer Kurzintelligenztest* (Hogrefe, Göttingen, 2015).
3. Turner, M. L. & Engle, R. W. Is working memory capacity task dependent? *J. Mem. Lang.* **28,** 127–154 (1989).
4. Liepmann, D., Beauducel, A., Brocke, B. & Amthauer, R. *I-S-T 2000 R: Intelligenz-Struktur-Test 2000 Revidiert* (Hogrefe, Göttingen, ed. 2, 2007).
5. Chan, J. C. K., Manley, K. D., Davis, S. D. & Szpunar, K. K. Testing potentiates new learning across a retention interval and a lag: A strategy change perspective. *J. Mem. Lang.* **102,** 83–96 (2018).
6. Kliegl, O. & Bäuml, K.-H. T. When retrieval practice promotes new learning – the critical role of study material. *J. Mem. Lang.* **120,** 104253 (2021).
7. Anderson, M. C., Bjork, R. A. & Bjork, E. L. Remembering can cause forgetting: retrieval dynamics in long-term memory. *J. Exp. Psychol. Learn.* **20,** 1063–1087 (1994).
8. Anderson, M. C. & Spellman, B. A. On the status of inhibitory mechanisms in cognition: memory retrieval as a model case. *Psychol. Rev.* **102,** 68–100 (1995).
9. Wallner, L. & Bäuml, K.-H. T. Beneficial effects of selective item repetition on the recall of other items. *J. Mem. Lang.* **95,** 159–172 (2017).
10. Bäuml, K.-H. T. & Trißl, L. Selective memory retrieval can revive forgotten memories. *Proc. Natl. Acad. Sci. U.S.A.* **119,** e2114377119 (2022).
